# Supplementary material for: Simultaneous and sensitive quantification of protein and low molecular weight persulfides, polysulfides and H2S in biological samples
Source: Nat Commun. 2025 Dec 4;17:85. doi: 10.1038/s41467-025-66795-5 (PMC12769671; doi:10.1038/s41467-025-66795-5)
Supplement: Supplementary file 1 — Supplementary Information [file 41467_2025_66795_MOESM1_ESM.pdf]

Supplementary material

Simultaneous and sensitive quantification of protein and low molecular weight persulfides, polysulfides and H_2S in biological samples

Jan Lj. Miljkovic[1], Nils Burger[1], Chak Shun Yu[1], Alexander H. Harkiss[2], Stefan Warrington[2], Stuart T. Caldwell[2], Scott A. Jones[1], Jordan J. Lee[3], Dunja Aksentijevic[4], Andrew M. James[1], Thomas Krieg[3], Richard C. Hartley[2*] & Michael P. Murphy[1,3*]

[1]Medical Research Council-Mitochondrial Biology Unit, University of Cambridge, Cambridge CB2 0XY, UK
[2]School of Chemistry, University of Glasgow, Glasgow, G12 8QQ, UK
[3] Department of Medicine, University of Cambridge, Cambridge CB2 0QQ, UK
[4]William Harvey Research Institute, Bart's and the London Faculty of Medicine and Dentistry, Queen Mary University of London, London, EC1M 6BQ, UK

[*]e-mail: mpm37@cam.ac.uk; Richard.Hartley@glasgow.ac.uk

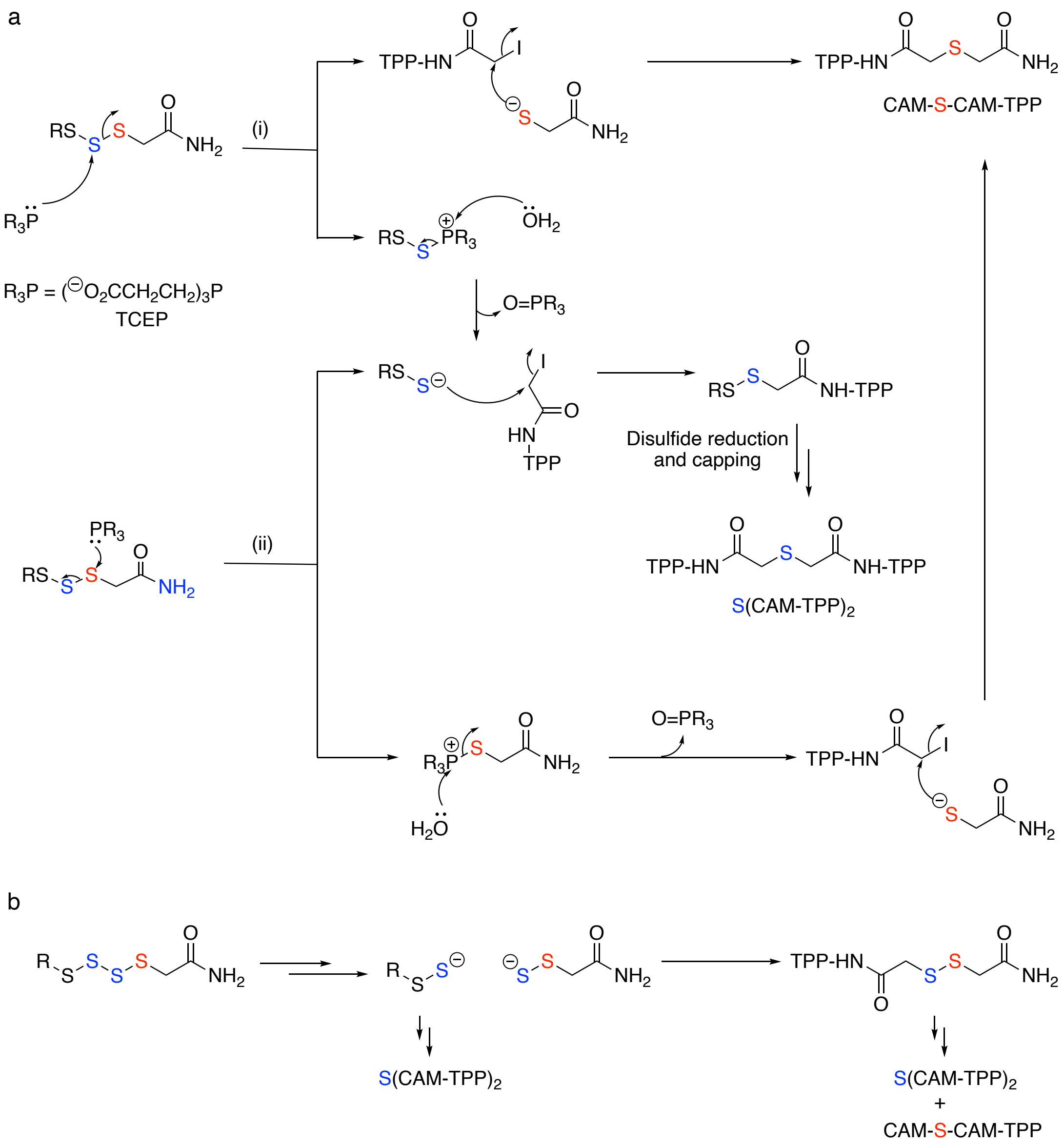

Supplementary Figure 1. Proposed mechanism for the specific production of TPP-CAM-S-CAM-TPP from sulfane sulfur atoms and CAM-S-CAM-TPP from the terminal sulfur atom after capping with IAM in the first step of procedure B. (**a**) Trisulfide example. The CAM-capped trisulfide can potentially be reduced by TCEP attack on the central sulfane sulfur atom or the terminal sulfur atom or the sulfur atom of the protein/ parent L_{MW}-SH. The processes involved in generation of $S(CAM\text{-}TPP)_2$ and CAM-S-CAM-TPP are illustrated by (i) TCEP attack on the internal sulfur breaking the S-S bond towards the terminal sulfur and (ii) TCEP attack on the terminal sulfur. Attack on the internal sulfur breaking the S-S bond towards the sulfur atom of the protein/parent L_{MW}-SH or direct attack on the latter sulfur atom give analogous processes. (**b**) A tetrasulfide example is used to show that breaking the bond between two sulfane sulfur atoms in a higher order polysulfide gives $S(CAM\text{-}TPP)_2$ from internal sulfane sulfur atoms and CAM-S-CAM-TPP from the terminal sulfur atom; alternative sites of attack are analogous to those shown in (**a**).

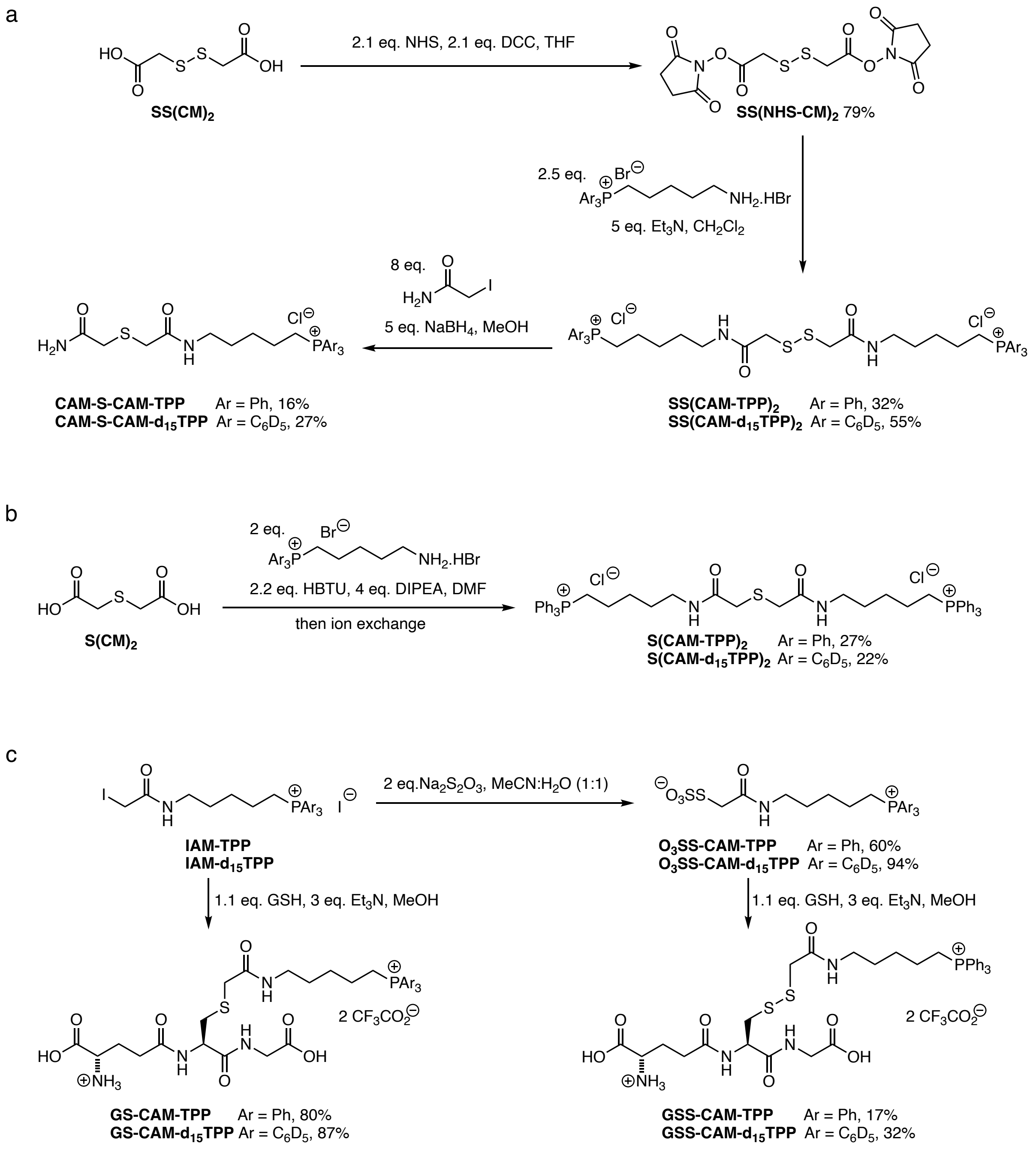

Supplementary Figure 2. Chemical syntheses. a, Synthesis of symmetrical disulfides and non-symmetrical sulfides. **b**, Synthesis of symmetrical sulfides. **c**, Synthesis of glutathione products.

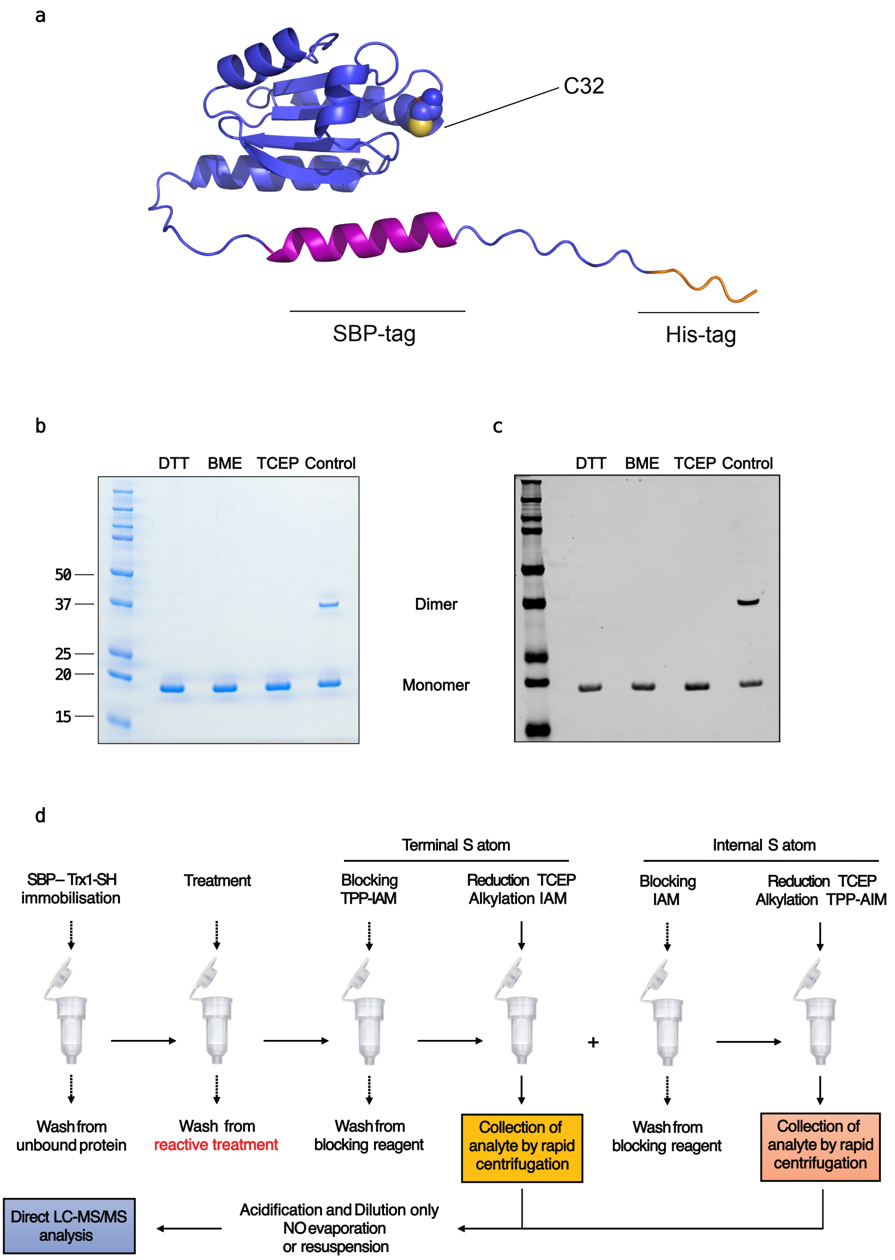

Supplementary Figure 3. Generation and analysis of Trx1. a, Structure of modified Trx1 showing the single Cys residue and the SBP and His tags. **b**, **c**, Purified Trx1 (170 µg) was incubated with 50 mM DTT, β-ME, TCEP or no reductant (Control) for 20 min at RT under argon, diluted in Laemmli's sample buffer and then 12 µg of protein was resolved by non-reducing 10% SDS-PAGE. Resolved proteins were either stained with Coomassie blue (**b**) or electroblotted on a nitrocellulose membrane and immunoprobed using a rabbit antibody against mouse Trx1 (**c**). **d**, Procedures for binding Trx1 to the NeutrAvidin beads, followed by treatment with Na_2S_2 or DTNB/Na_2S, washing, and subsequent analysis to generate CAM-S-CAM-TPP by procedure A and $S(CAM\text{-}TPP)_2$ by procedure B are shown. Selected elements in Supplementary Fig. 3d were prepared with the assistance of BioRender software. Source data are provided as a Source Data file.

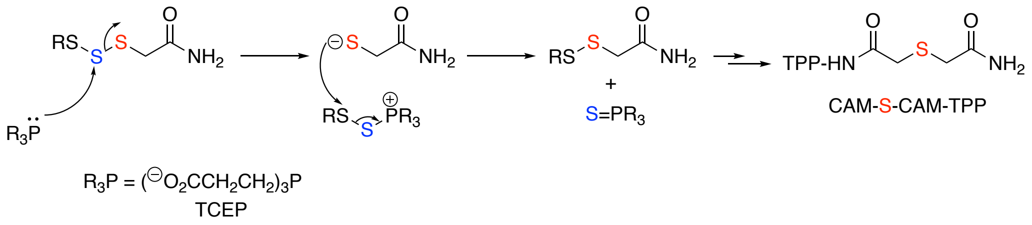

Supplementary Figure 4. Mechanism of potential under-reporting of internal sulfur(0) of polysulfides in absence of trapping by IAM-TPP. TCEP attack on the internal sulfur(0) generates a thiolate and a phosphonium ion; if the thiolate is not capped by IAM-TPP, it could recombine with the phosphonium fragment to generate TCEP=S, removing the internal sulfur from detection.

a

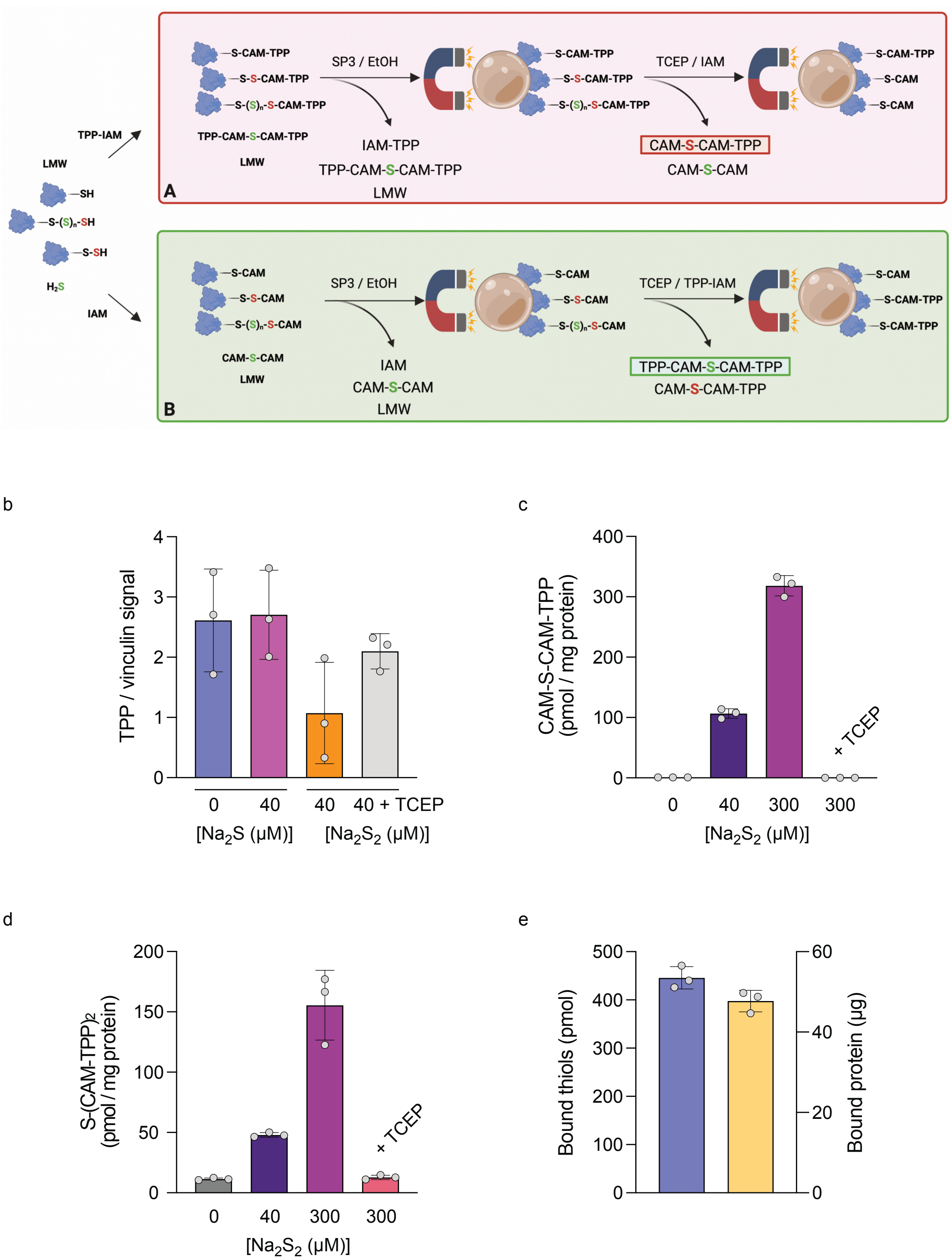

Supplementary Figure 5. Assessment of protein persulfides and polysulfides. a, Schematic of immobilisation of protein extracts on SP3 beads and subsequent analysis. **b**, HEK 293 cell lysates were exposed to Na_2S (40 µM) or Na_2S_2 (40 µM) for 7 minutes, alkylated with IAM-TPP, bound to the SP3 beads, washed, eluted, resolved by SDS-PAGE and immunoprobed for TPP and vinculin. The graph shows quantification of the TPP signal relative to that of vinculin **c**, **d** Quantification of protein terminal thiolate of persulfides and protein persulfudes by procedure A (**c**, as CAM-S-CAM-TPP) and internal sulfane sulfur of protein polysulfides by

procedure B (**d**, as S(CAM-TPP)$_2$) from rat heart tissue lysates obtained by exposing tissue lysates (100 μg wet weight) to Na_2S_2 (40 and 300 μM) for 7 min. After alkylation samples were immobilised on SP3 beads, washed, reduced with TCEP and re-alkylated. Analytes were eluted from the SP3 beads and analysed by LC-MS/MS. **e**, Protein thiol determination from rat heart native tissue lysate using Ellman's reagent. Concentration of protein was determined using the BCA assay. Protein and thiol concentration was normalised to the amount of SP3 slurry used in each sample (2 mg). Data are means ± S.D., n = 3 (n represent a single technical replicate). Selected protein structure elements in Figure 5a were prepared with the assistance of BioRender software. Source data are provided as a Source Data file.

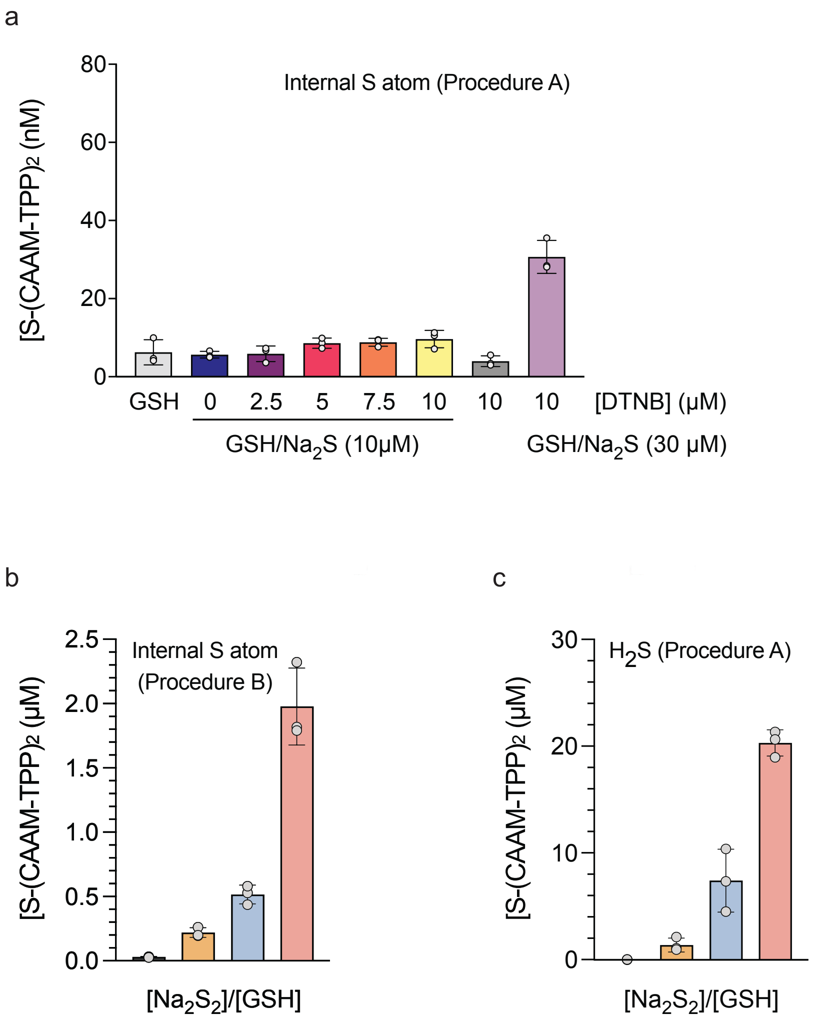

Supplementary Figure 6. Assessment of LMW persulfides. a, GSH (10 μM) was incubated with DTNB (0 - 10 μM) and then reacted with Na_2S (0, 10, 30 μM). **a.** Samples were then processed by Procedure B and S(CAM-TPP)$_2$ quantified. **b**, GSH (100 μM) was incubated with Na_2S_2 (0, 100, 200 and 300 μM) and processed by procedure B to quantify S(CAM-TPP)$_2$. **c**, GSH (100 μM) was incubated as in **c** and processed by Procedure A and S(CAM-TPP)$_2$ quantified. Data are mean ± SD, n = 3 (n represent a single technical replicate). Source data are provided as a Source Data file.

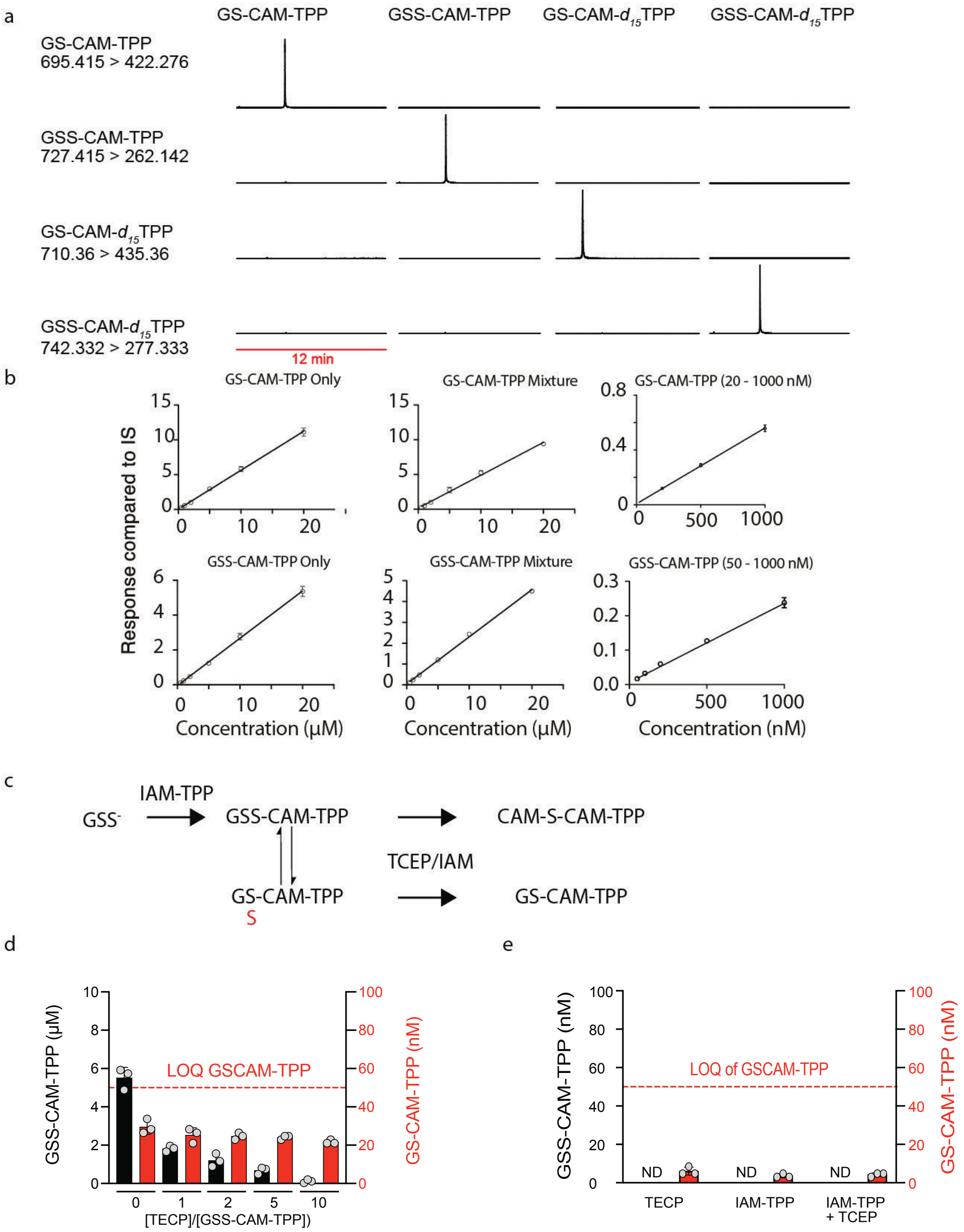

Supplementary Figure 7. LC-MS/MS quantification of GS-CAM-TPP and GSS-CAM-TPP. **a**, MRM chromatograms analysing 200 pmol GS-CAM-TPP, GSS-CAM-TPP and their deuterated internal standards (IS), with diagnostic m/z transitions indicated. **b**, LC-MS/MS standard curves for GS-CAM-TPP and GSS-CAM-TPP. MS response was normalised to 25 μM and 5 μM deuterated IS, respectively. **c**, Possible isomerisation and desulfuration of a persulfide after reaction with IAM-TPP. **d**, GSS-CAM-TPP (5 μM) was incubated with TCEP (0 - 100 μM) and GS-CAM-TPP and GSS-CAM-TPP levels were quantified as in **b. e**, TCEP (100 μM), IAM-TPP (10 mM) or IAM-TPP and TCEP (10 mM) were incubated for 30 min and GS-CAM-TPP and GSS-CAM-TPP quantified by LC-MS/MS. LoD for GS-CAM-TPP under these conditions is 50 nM. ND = not detected. Data are mean ± S.D., n = 3 (n represent a single technical replicate; Supplementary fig 7d, 7e). Source data are provided as a Source Data file.

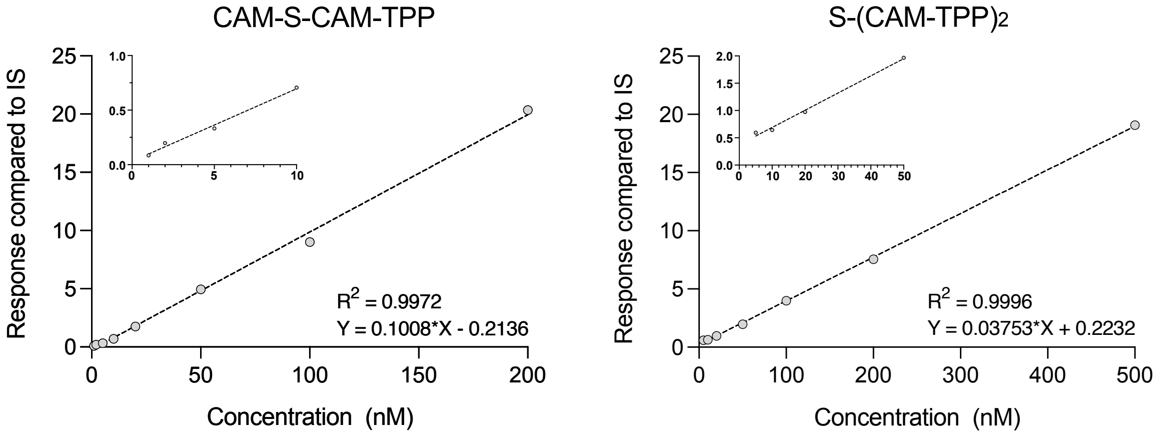

Supplementary Figure 8. LC-MS/MS quantification of CAM-S-CAM-TPP and S(CAM-TPP)$_2$. Matrix-matching calibration curves (containing IAM-TPP, TCEP and IAM) for the detection of CAM-S-CAM-TPP and S(CAM-TPP)$_2$ by LC-MS/MS. The MS response at different compound concentrations was normalised to that of 50 nM of the corresponding deuterated internal standard. Source data are provided as a Source Data file.

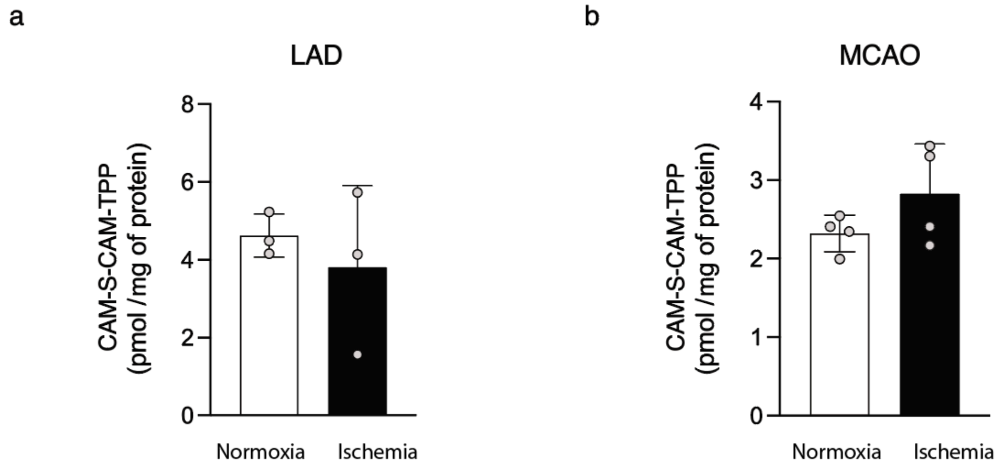

Supplementary Figure 9. Comparison of protein persulfidation in mouse heart and brain tissues under ischemic and control conditions using *in vivo* experimental models. Snap-frozen heart and brain tissue samples were collected following *in vivo* ischemia models and processed using Procedure A, adapted to SP3 bead-based enrichment. Protein persulfides (CAM-S-CAM-TPP) were quantified and normalized to total protein eluted from SP3 beads for each organ. a, Comparative profiling of protein persulfidation in mouse heart tissue from control regions (non-risk area) versus ischemic regions (area-at-risk) following left anterior descending (LAD) coronary artery ligation. b. Protein persulfidation analysis in brain tissue from mice subjected to middle cerebral artery occlusion (MCAO), comparing the ischemic hemisphere (ipsilateral) to the non-ischemic control hemisphere (contralateral). All data are presented as mean ± S.D., n = 3, Supplementary Fig 9a or n = 4, Supplementary Fig 9b (n represent a single biological replicate). Source data are provided as a Source Data file.

Unprocessed original 10% SDS PAGE of purified TRx1

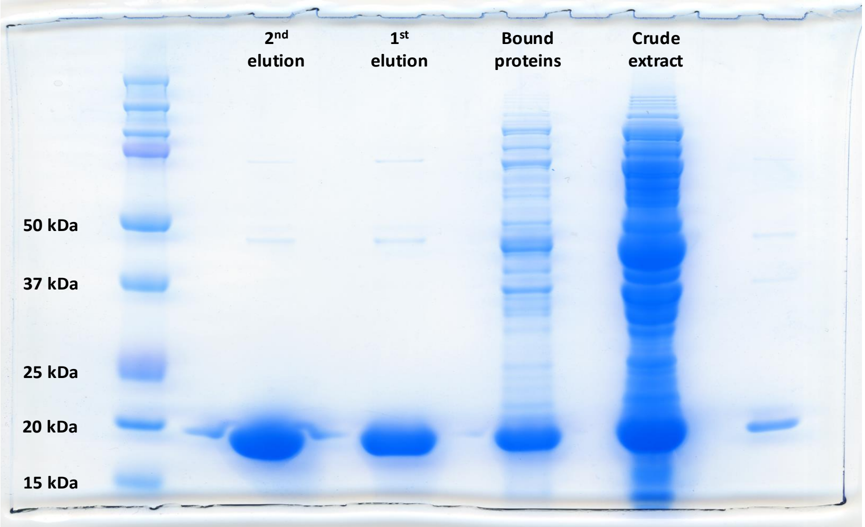

Supplementary Figure 10. Unprocessed original SDS–PAGE gel illustrating the purification procedure of Trx1 used in this study.

Supplementary Table 1. Summary of the expected and detected masses for CAM-S-CAM-TPP, $S(CAM\text{-}TPP)_2$, and their corresponding deuterated analogues.

Compound	Expected mass	Detected Mass
CAM-S-CAM-TPP	479.1917	479.13
CAM-S-CAM-d_{15}TPP	494.2859	494.38
$S(CAM\text{-}TPP)_2$	405.1764	405.45
S(CAM-d_{15}TPP)$_2$	420.2705	420.40

Supplementary Table 2. Summary of the HPLC gradient conditions used for the analysis of CAM-S-CAM-TPP and $S(CAM\text{-}TPP)_2$.

Time (min)	%A	%B
0-0.3	95	5
0.3-2	0	100
2-2.5	0	100
2.5-2.8	95	5
2.8-3	95	5

Supplementary Table 3. Summary of the MS/MS transitions used for the quantification of CAM-TPP, $S(CAM\text{-}TPP)_2$, and their corresponding deuterated analogues.

Compound	Mol. weight	Charge	Transition (m/z)	Cone voltage (V)	Collision energy (V)
CAM-S-CAM-TPP	479.1917	(M^{+})	479.2660 > 420.2314	66	32
CAM-S-CAM-d_{15}TPP	494.2859	(M^{+})	494.3299 > 435.3634	4	34
$S(CAM\text{-}TPP)_2$	405.1764	(M^{+2})	405.3298 > 262.2027	62	32
S(CAM-d_{15}TPP)$_2$	420.2705	(M^{+2})	420.3937 > 277.4056	28	34

Supplementary Table 4. Summary of the modified HPLC gradient used to prepare standard curves for the analysis of CAM-S-CAM-TPP and $S(CAM\text{-}TPP)_2$ from Langendorff experiments.

Time (min)	%A	%B
0-0.3	100	0
0.3-5	80	20
5-6	80	20
6-7	50	50
7-9	50	50
9-10	0	100
10-12	0	100
12-15	100	0

Supplementary Table 5. Summary of the expected and detected masses of GS-TPP, GSS-TPP, and their corresponding deuterated analogues.

Compound	Expected mass	Detected Mass
GS-CAM-TPP	695.2663	695
GS-CAM-d_{15}TPP	710.3604	710
GSS-CAM-TPP	727.2384	727
GSS-CAM-d_{15}TPP	742.3325	742

Supplementary Table 6. Summary of the HPLC gradient used to analyse GS-TPP, GSS-TPP, and their corresponding deuterated analogues.

Time (min)	%A	%B
0	95	5
2	95	5
8	5	95
9	5	95
10	95	5
12	95	5

Supplementary Table 7. Summary of the MS/MS transitions used for the quantification of GS-TPP, GSS-TPP, and their corresponding deuterated analogues.

Compound name	Mol. weight	Charge	Transition (m/z)	Cone energy (V)	Collision energy (V)
GS-CAM-TPP	695.2663	(M^+)	695>422	38	40
GS-CAM-d_{15}TPP	710.3604	(M^+)	710>435	127	50
GSS-CAM-TPP	727.2384	(M^+)	727>262	98	40
GSS-CAM-d_{15}TPP	742.3325	(M^+)	742>277	127	50
