# Supplementary material for: Simultaneous and sensitive quantification of protein and low molecular weight persulfides, polysulfides and H2S in biological samples
Source: Nat Commun. 2025 Dec 4;17:85. doi: 10.1038/s41467-025-66795-5 (PMC12769671; doi:10.1038/s41467-025-66795-5)
Supplement: Supplementary file 2 — Reporting Summary [file 41467_2025_66795_MOESM2_ESM.pdf]

nature portfolio

Corresponding author(s): Michael P. Murphy

Last updated by author(s): Nov 10, 2025

Reporting Summary

Nature Portfolio wishes to improve the reproducibility of the work that we publish. This form provides structure for consistency and transparency in reporting. For further information on Nature Portfolio policies, see our Editorial Policies and the Editorial Policy Checklist.

Statistics

For all statistical analyses, confirm that the following items are present in the figure legend, table legend, main text, or Methods section.

n/a	Confirmed	
☒	☐	The exact sample size (*n*) for each experimental group/condition, given as a discrete number and unit of measurement
☒	☐	A statement on whether measurements were taken from distinct samples or whether the same sample was measured repeatedly
☒	☐	The statistical test(s) used AND whether they are one- or two-sided<br>*Only common tests should be described solely by name; describe more complex techniques in the Methods section.*
☒	☐	A description of all covariates tested
☒	☐	A description of any assumptions or corrections, such as tests of normality and adjustment for multiple comparisons
☒	☐	A full description of the statistical parameters including central tendency (e.g. means) or other basic estimates (e.g. regression coefficient) AND variation (e.g. standard deviation) or associated estimates of uncertainty (e.g. confidence intervals)
☒	☐	For null hypothesis testing, the test statistic (e.g. *F*, *t*, *r*) with confidence intervals, effect sizes, degrees of freedom and *P* value noted<br>*Give P values as exact values whenever suitable.*
☒	☐	For Bayesian analysis, information on the choice of priors and Markov chain Monte Carlo settings
☒	☐	For hierarchical and complex designs, identification of the appropriate level for tests and full reporting of outcomes
☒	☐	Estimates of effect sizes (e.g. Cohen's *d*, Pearson's *r*), indicating how they were calculated

Our web collection on statistics for biologists contains articles on many of the points above.

Software and code

Policy information about availability of computer code

Data collection	The integration of the peak area for CAM-S-CAM-TPP, S(CAM-TPP)2, GS-CAM-TPP, GSS-CAM-TPP and their corresponding deuterated analytical standards (CAM-S-CAMd15-TPP, S(CAM-TPPd15)2, GS-CAMd15-TPP and GSS-CAMd15-TPP) was performed using MassLynx 4.1 software. The data reflecting the formation of GS-TNB adduct were acquired using Olis GlobalWorks software. NMR- TopSpin v 3.6 or 4.4. Mass Spectrometry Agilent MassHunter Workstation, LC/MS Data Aquisition (version 10.1)
Data analysis	Protein bands from PAGE gel and western blot experiments were analysed using Image Studio lite 2.5 (LI-COR). Statistical analysis was performed using Prism 10 (Graphpad). NMR data were analyzed using MestReNova v14.3.0-30573.

For manuscripts utilizing custom algorithms or software that are central to the research but not yet described in published literature, software must be made available to editors and reviewers. We strongly encourage code deposition in a community repository (e.g. GitHub). See the Nature Portfolio guidelines for submitting code & software for further information.

Data

Policy information about availability of data

All manuscripts must include a data availability statement. This statement should provide the following information, where applicable:

- Accession codes, unique identifiers, or web links for publicly available datasets
- A description of any restrictions on data availability
- For clinical datasets or third party data, please ensure that the statement adheres to our policy

All raw and processed NMR data had been deposited. https://doi.org/10.5525/gla.researchdata.1879
All raw and unprocessed LC-MS data had been deposited. https://doi.org/10.5061/dryad.7m0cfxq8k

Research involving human participants, their data, or biological material

Policy information about studies with human participants or human data. See also policy information about sex, gender (identity/presentation), and sexual orientation and race, ethnicity and racism.

Reporting on sex and gender	N/A
Reporting on race, ethnicity, or other socially relevant groupings	N/A
Population characteristics	N/A
Recruitment	N/A
Ethics oversight	N/A

Note that full information on the approval of the study protocol must also be provided in the manuscript.

Field-specific reporting

Please select the one below that is the best fit for your research. If you are not sure, read the appropriate sections before making your selection.

☒ Life sciences ☐ Behavioural & social sciences ☐ Ecological, evolutionary & environmental sciences

For a reference copy of the document with all sections, see nature.com/documents/nr-reporting-summary-flat.pdf

Life sciences study design

All studies must disclose on these points even when the disclosure is negative.

Sample size	A minimum of 3 biological replicates per experiment was used to achieve biological variability.
Data exclusions	Data were not excluded from the study.
Replication	The number of biological replicates is indicated in the figure legend.
Randomization	All samples, including for in vivo and for ex vivo experiments were randomly divided into groups for analysis.
Blinding	Due to the absence of experimental treatment, blinding was not applied.

Reporting for specific materials, systems and methods

We require information from authors about some types of materials, experimental systems and methods used in many studies. Here, indicate whether each material, system or method listed is relevant to your study. If you are not sure if a list item applies to your research, read the appropriate section before selecting a response.

Materials & experimental systems

n/a	Involved in the study	
☐	☒	Antibodies
☐	☒	Eukaryotic cell lines
☒	☐	Palaeontology and archaeology
☐	☒	Animals and other organisms
☒	☐	Clinical data
☒	☐	Dual use research of concern
☒	☐	Plants

Methods

n/a	Involved in the study	
☒	☐	ChIP-seq
☒	☐	Flow cytometry
☒	☐	MRI-based neuroimaging

Antibodies

Antibodies used

Primary mouse monoclonal antibody (clone 3A1; ab 16965) against human Thioredoxin 1 protein was purchased from Abcam, UK and it was used at 1: 1000 working dilution. To detect CAM-TPP-based alkylated proteins, rabbit purified antiserum against TPP was used at 1: 1000 working dilution. For detection of western blot signals obtained by primary antibodies, IRDye® 680RD Goat anti-Mouse IgG Secondary Antibody (LiCOR; # 926-68070) and IRDye® 800CW Goat anti-Rabbit IgG Secondary Antibody, (LiCOR; # 926-32211) were used at 1 : 15 000 working dilution.

Validation

Mouse monoclonal antibody against human Thioredoxin 1 protein was used and described in several publications available on the vendor (Abcam) website. The rabbit antiserum against TPP was validated in the following publication: Aparna Venkatraman, Aimee Landar, Ashley J. Davis, Elena Ulasova, Grier Page, Michael P. Murphy, Victor Darley-Usmar, and Shannon M. Bailey. Oxidative modification of hepatic mitochondria protein thiols: effect of chronic alcohol consumption. American Journal of Physiology-Gastrointestinal and Liver Physiology 2004 286:4, G521-G527.

Eukaryotic cell lines

Policy information about cell lines and Sex and Gender in Research

Cell line source(s)

The human HEK-293 cell line was purchased from American Type Culture Collection (ATCC).

Authentication

The cell line was characterized previously: Graham FL, et al. Characteristics of a human cell line transformed by DNA from human adenovirus type 5. J. Gen. Virol. 36: 59-72, 1977. PubMed: 886304.

Mycoplasma contamination

The cell line was tested negative for mycoplasma contamination.

Commonly misidentified lines
(See ICLAC register)

N/A

Animals and other research organisms

Policy information about studies involving animals; ARRIVE guidelines recommended for reporting animal research, and Sex and Gender in Research

Laboratory animals

Wildtype (WT) mice (C57/BL/6/J) and wildtype (WT) rats (Wistar rats) were purchased from Charles River Laboratories, UK.

Wild animals

This study did not involve wild animals.

Reporting on sex

Hearts used in Langendorff perfusion experiments were obtained from male WT (C57BL/6J; Charles River Laboratories, UK) mice. Rat tissues from various organs were obtained from male WT Wistar rats (Charles River Laboratories, UK).

Field-collected samples

This study did not involve field-collected samples.

Ethics oversight

All animal experiments were carried out in accordance with the UK Animals (Scientific Procedure) Act, 1986 (Home Office PPL np. P6C97520A and PP1730969). All procedures were approved by the University of Cambridge Animal welfare and ethical review Body (AWERB) Committee.

Note that full information on the approval of the study protocol must also be provided in the manuscript.

Plants

Seed stocks	N/A
Novel plant genotypes	N/A
Authentication	N/A
