# Supplementary figures and images for: Simultaneous and sensitive quantification of protein and low molecular weight persulfides, polysulfides and H2S in biological samples
Source: Nat Commun. 2025 Dec 4;17:85. doi: 10.1038/s41467-025-66795-5 (PMC12769671; doi:10.1038/s41467-025-66795-5)

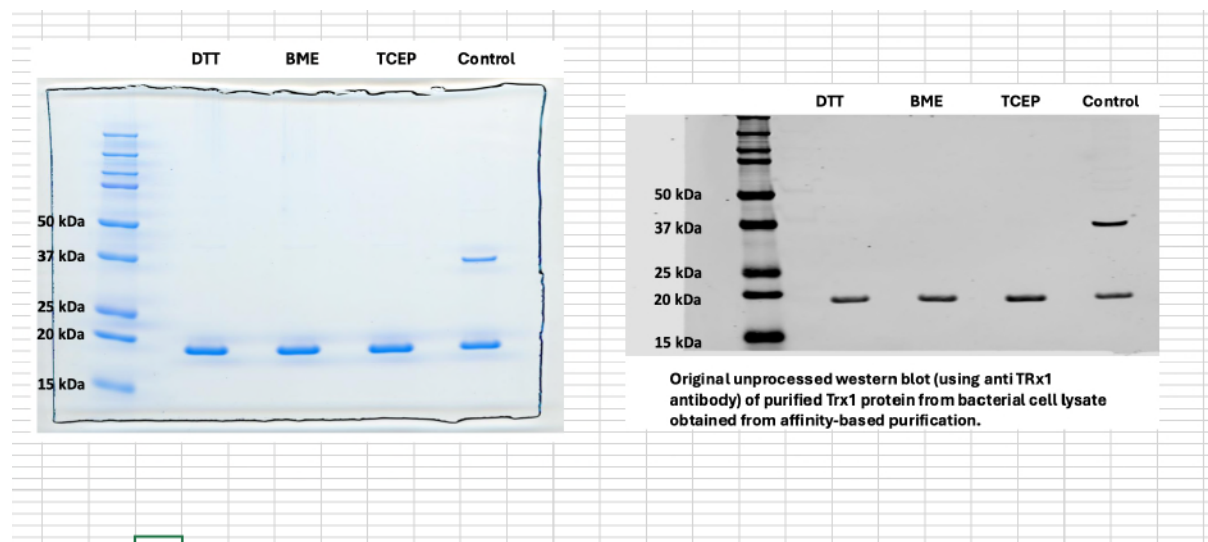

Uncropped gels for Supplementary Fig 3

Supplement: Supplementary file 4 — Source Data [file 41467_2025_66795_MOESM4_ESM.zip › 2.pdf]
